# Long-term follow-up of self-expandable metallic stents in benign tracheobronchial stenosis: a retrospective study

**DOI:** 10.1186/s12890-019-0793-y

**Published:** 2019-02-08

**Authors:** Xiao-feng Xiong, Li Xu, Li-li Fan, De-yun Cheng, Bi-xia Zheng

**Affiliations:** 1 Department of Respiratory and Critical Care Medicine, West China Hospital of Sichuan University, NO.37 Guoxue Alley, Chengdu, 610041 Sichuan China; 2 Department of Respiratory Medicine, Chengdu Third People’s Hospital, NO.82 Qinglong Street, Qingyang District, Chengdu, 610031 Sichuan China

**Keywords:** Benign tracheobronchial stenosis, Self-expandable metallic stents, Airway stenting

## Abstract

**Background:**

Self-expandable metallic stents (SEMSs) have enabled a approving management of malignant airway stenosis. However, the long-term efficacy and safety of this treatment in patients with benign airway stricture are unclear. We conducted this study to retrospectively determine the efficacy and long-term outcomes in patients who have undergone SEMS placement for benign tracheobronchial stenosis.

**Methods:**

All patients treated with SEMSs from July 2003 to June 2016 were reviewed for symptomatic response, complications, and long-term outcomes.

**Results:**

Total 131 stents were successfully deployed in 116 patients. Ninety-eight patients demonstrated clinical improvement after stent insertion (84.48%; 95% confidence interval [CI]: 77.89–91.07). Compared with uncovered stents, covered stents were associated with more sore throats complaints or chest pain (13.89% versus 28.81%, *P* = 0.036) and with higher incidences of major and minor granulation tissue formation and with recurrent stenosis (4.17% versus 15.25%, *P* = 0.029; 11.11% versus 37.29%, *P* < 0.0001 and 9.72% versus 28.81%, *P* = 0.005, respectively). Each covered and uncovered stent developing tissue hyperplasia required a median of 2 (range: 1–15) and 1(range: 1–7) fibrobronchoscope with electrocautery therapy, respectively. At follow-up (median: 1276 days; range: 2–4263), 68 patients had complete resolution, 15 remained under interventional treatment, 8 had bronchial occlusions, 7 underwent surgery, 14 were lost to follow-up, and 4 died of stent unrelated causes.

**Conclusion:**

SEMS placement achieved most clinical improvement among patients in our study, if adequate endotracheal measures were used to address stent-related complications. The use of permanent SEMSs for benign tracheobronchial stenosis was effective and safe for the majority of patients in a long-term follow-up.

**Trial registration:**

The study has been retrospectively registered in the China Clinical Trial Registry on October 21, 2018 (Registry ID: ChiCTR1800019024).

## Background

Airway obstruction is potentially life-threatening. Corrective therapies to guarantee a sufficient airstream are essential for patients with severe tracheobronchial obstructions. There are several surgical and nonsurgical therapies available in clinic. A cutting-edge nonsurgical therapy is bronchoscopic airway stenting. Tracheal stenting for symptomatic stenosis is reserved for patients with lesions that are deemed unresectable, due to local or general conditions [1]. Treatment for benign tracheobronchial stenosis treated with stenting includes post-inflammatory stenosis (after tuberculosis or airway fungal disease), post-traumatic stenosis (after intubation or tracheostomy), post-anastomotic stenosis (after lung transplantation), and extrinsic compression [2].

The use of self-expandable metallic stents (SEMSs) has become a common procedure, mainly in malignant tracheobronchial stenosis. For benign tracheobronchial lesions, silicone stents are preferred. Previously, however, silicone stents were not available in some developing countries. Thus, some interventional pulmonologists have also used SEMSs in benign tracheal stenosis, and there is still controversy with respect to the long-term patency and complications of this therapy. Moreover, due to the lack of comprehensive observations on the efficacy and long-term outcomes this treatment remains controversial. This procedure has been performed at our institution for nearly 13 years in a variety of benign tracheal stenosis procedures, and approximately 10 years of follow-up data are now available. Thus, the purpose of this study was to retrospectively determine the efficacy and long-term outcomes in patients who have undergone tracheobronchial metal stent placement for benign diseases.

## Methods

### Design

This research was a retrospective study. It identified all patients with benign tracheal stenosis who underwent SEMS placement from July 2003 to June 2016 at the Respiratory and Critical Care Medicine Unit of West China Hospital (Chengdu, China). This study was approved by the Institutional Review Board of West China Hospital of Sichuan University. Verbal consent was obtained from the patients who responded. Patients’ clinical archives were reviewed to collect the information on sex, age, type and site of airway strictures, associated diseases, outcomes after SEMS placement, and bronchoscope follow-up. Stents were evaluated according to type, further interventional therapy, complications, and longevity in the airway.

### Stent implantation

SEMS (Micro-Tech [Nanjing] Co., Ltd., Nanjing, Peoples Republic of China), a self-expandable metallic stent made entirely by nickel-titanium alloy, was used in our research. Each patient had a chest computed tomography (CT) scan and flexible bronchoscopy prior to SEMS placement to evaluate the exact position of the stenotic section, its length, and its relation to significant anatomical landmarks such as the vocal cords and the tracheal bifurcation [3]. Under conscious sedation and local anesthesia, the procedure of SEMS placement and the evaluation of stent condition were performed by flexible bronchoscopy as previously described [4–6]. The procedure of stent deployment is as follows. The bronchoscope is inserted first unilaterally through a nostril into the trachea and is navigated to the proximal end of the lesion. A guide wire is inserted via the bronchoscope and passed through the stenotic segment. The bronchoscope is withdrawn, leaving the guide wire at the lesion site. Under bronchoscopic visualisation, the delivery catheter is advanced over the guide wire to deploy the stent. The delivery catheter, guide wire and bronchoscope are then withdrawn, leaving the stent in the lesion site [6].

Stent types (diameter, length, shape, covered or uncovered) were selected according to patient’s condition, bronchoscopic image and physician’s experience. Uncovered stents were usually selected for the treatment of benign diseases to relieve symptoms, especially in the bronchial airway. Covered stents were usually used for patients with benign airway stenosis to improve feasibility of stent removal. In areas where covered stents could obstruct other bronchial airways, uncovered stents were used.

### Follow-up and study end points

The follow-up included a first bronchoscopy examination 1–3 days after stent deployment, to check the position and patency of the stent. Further follow-up examinations were conducted if the patient appeared recurrent signs of airway stenosis. If the post-interventional course was uneventful, patients were follow-up every 3 months. Bronchoscopy was also applied just before and after stents were removed, or when complications occurred.

Technical success was defined as a successful deployment of the stent without any major complications. Situations when the patient showed a marked improvement in respiratory symptoms, when the patient was weaned off mechanical ventilation, or when the patient experienced a reduction in the rate of respiratory infections in the subsequent period after stent placement (on average 2–3 days) were considered clinical improvements. Recurrent stenosis was defined as the reappearance of symptoms and identifiable airway restenosis on bronchoscopy. Complete resolution was considered accomplished when the tracheobronchial lumen maintained > 70% of its diameter after covered stent removal or when the SEMS did not require further calibrations and became completely epithelialized and free from granulation tissue. An increase in sputum retention diagnosed when it became symptomatic and increased after stent deployment was considered a complication. Patients were considered lost to follow-up when they were not reachable by telephone for at least one year after the last examination. The definition of the terms was based on the previous study [7].

Stent removal was indicated when either the stent was no longer required or stent-related complications were observed, such as stent migration or notable tissue hyperplasia around the stent. Stents retrieval was also accomplished by flexible bronchoscopy.

### Statistical analyses

All statistical analyses were conducted using SPSS Version 20.0(IBM Corp, Chicago, IL, USA), and a *P* value < 0.05 was considered statistically significant. We divided the study population into two groups according to the type of stent or the cause of stricture. Continuous data were used the Student’s *t* test, and categoric values between those with covered stents and those with uncovered stents were compared using the chi-square or Fisher’s exact test. Complications were compared between the two groups using Pearson’s *χ*^*2*^ test or Fisher’s exact test. Stent survival time and time-to-granulation tissue formation in two groups were evaluated by Kaplan-Meier analysis, and survival curves were compared using log-rank test. Confidence intervals (CIs) were calculated using exact likelihood. Mean ± standard deviation or median (range) were used to express quantitative variables.

## Results

### Population

The study identified 116 patients (57 men and 59 women; age range: 17–74 years; mean age: 38.96 years) affected by benign tracheal stenosis resulting from tuberculosis (*n* = 55), percutaneous dilation tracheotomy (*n* = 37), endotracheal tube (*n* = 16), postbronchial repair (*n* = 3), relapsing polychondritis (*n* = 2), fungal infection (*n* = 1), tracheoesophageal fistula repair (*n* = 1), and mediastinal cyst (*n* = 1). All 116 patients experienced severe dyspnea or potential life-threatening tracheal obstruction (Table 1).

**Table 1:** The cause and site of stricture, clinical result after stenting, definitive outcomes are described

Cause of stricture	Site of stricture	Patients (*n*)	Covered stents (*n*)	Clinical result after stenting	Definitive outcomes
Post-inflammatory stenosis					
Tuberculosis	UT	2	0	Clinical improved	Resolution
	MT	1	1	Clinical improved	Resolution
	LT	1	0	Clinical improved	Dead
	MT + RMSB	1	1	Clinical improved	Under treatment
	RMLB	5	3	4 Clinical improved; 1 not improved	2 Resolution;2 bronchial occlusion;1 Under treatment
	RMSB	9	2	8 Clinical improved; 1 not improved	6 Resolution,1bronchial occlusion;2 lost to follow-up
	LMSB	36	13	28 Clinical improved; 8 not improved	25 Resolution,5 bronchial occlusion;3 surgery;3 lost to follow-up
Fungal infection	UT + MT	1	1	Not improved	Under treatment
Post-traumatic stenosis					
Tracheotomy	UT	27	13	25 Clinical improved; 2 not improved	14 Resolution,1 surgery,6 under treatment;2 dead;4 lost to follow-up
	UT + MT	4	3	2 Clinical improved; 2 not improved	2 Resolution,1 surgery,1 dead
	MT	5	3	3 Clinical improved; 2 not improved	2 Resolution,1 surgery,1 under treatment;1 lost to follow-up
	RMSB	1	1	Clinical improved	Surgery
Endotracheal tube	UT	13	5	12 Clinical improved; 1 not improved	7 Resolution,4 under treatment;2 lost to follow-up
	MT	3	1	Clinical improved	2 Resolution,1 under treatment
Bronchial repair	RMSB	2	1	Clinical improved	1 Resolution,1 surgery,1 lost to follow-up
	LMSB	1	1	Clinical improved	Resolution
Tracheoesophageal fistula repair	UT	1	0	Clinical improved	Resolution
Relapsing polychondritis	LT + LMSB+RMSB	1	0	Clinical improved	Lost to follow-up
	MT + LT	1	0	Clinical improved	Resolution
Mediastinal cyst	UT + MT	1	1	Clinical improved	Resolution

Abbreviations: *UT* upper trachea, *MT* meddle trachea, *LT* lower trachea, *RMSB* right main stem bronchus, *RMLB* right middle lobar bronchus, *LMSB* left main stem bronchus

### Stent position

We totally placed 131 stents in 116 patients. Characteristics of patients and stents are summarized in Table 1. All stents were straight except for a bifurcation (Y) stent for one patient with relapsing polychondritis. In terms of stent position, 60 patients were inserted stents in the trachea, 54 in the bronchus, and 2 in both sites. Tracheal stents were deployed in the upper trachea (*n* = 43; 71.66%), middle trachea (*n* = 9; 15.00%), lower trachea (*n* = 1; 1.67%), upper and middle trachea (*n* = 6; 10.00%), and middle and lower trachea (*n* = 1; 1.67%). Bronchial stents were deployed in the left main stem bronchus (*n* = 37; 68.52%), right main stem bronchus (*n* = 12; 22.22%), and right middle lobar bronchus (*n* = 5; 9.26%). Fifteen patients underwent stent implantation again.

### Stent management

#### Covered nitinol stents

Each stent developing granulation tissue (*n* = 32) required a median of 2 (range: 1–15) rounds of fibrobronchoscope with electrocautery therapy. Of the 59 covered nitinol stents, 52 (88%) were removed or substituted a median of 32 days (range: 2–142 days) after placement. Of the 52 removed stents, 37 were removed because patients’ conditions had improved, and the stents were no longer needed; 9 were removed because of obstructing granulation tissue; 3 were removed because of dislocation; and 7 stents were removed and replaced with the larger ones, 5 with uncovered nitinol stents and 2 with silicone stents. Complications related to stent removal included mucosal edema (15%) and respiratory distress (4%) that were successfully treated by medical therapy.

#### Uncovered nitinol stents

**Fig. 1:**
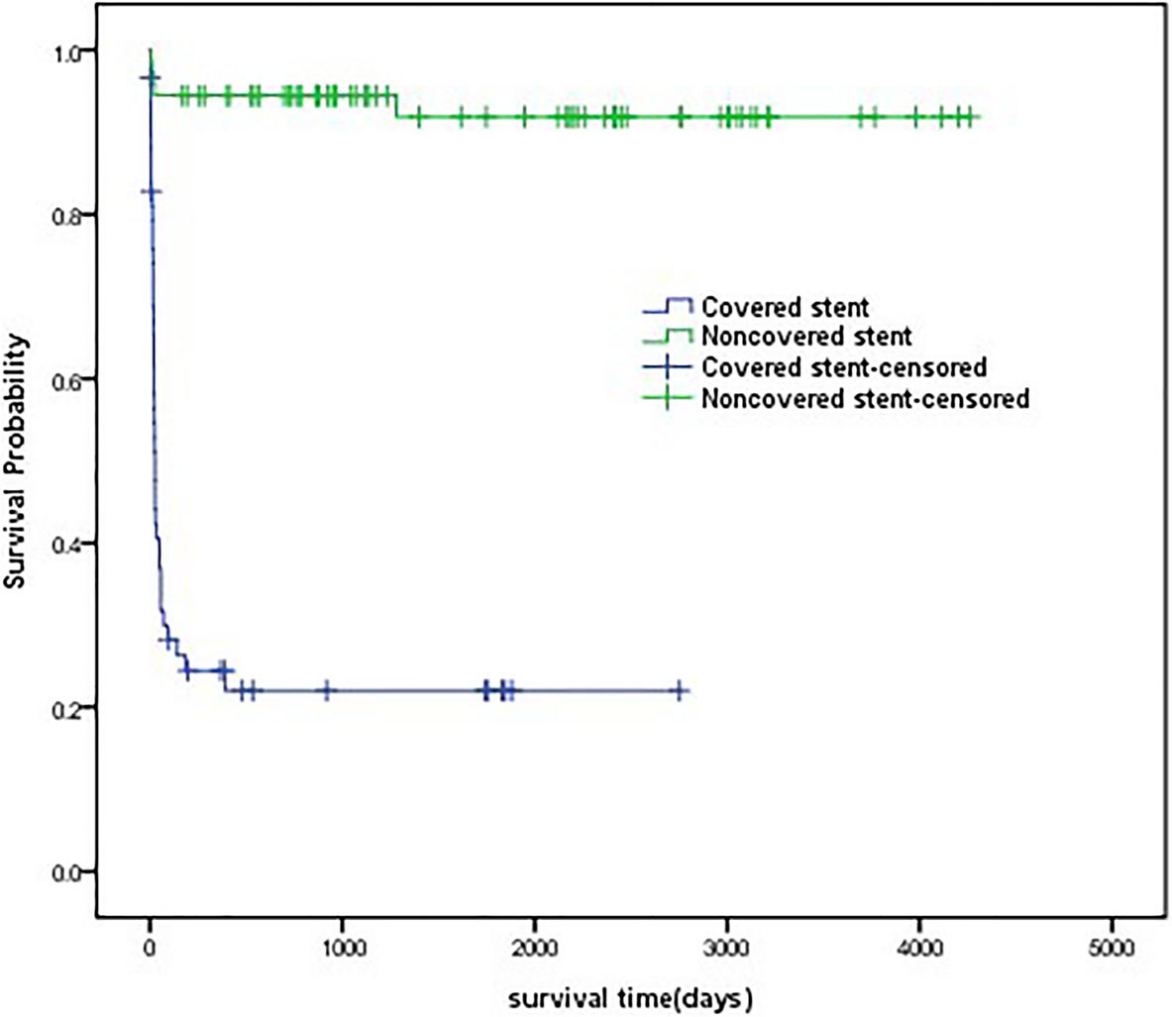
Stent survival curves Kaplan-Meier curves showing survival time for covered and uncovered stents implantation in patients with benign tracheobronchial stenosis (median 25 days vs 1339 days, *P* < 0.0001)

Each stent developing granulation tissue (*n* = 9) required a median of 1 (range: 1–7) round of fibrobronchoscope with electrocautery therapy. Of the 72 uncovered nitinol stents, 3 stents were removed a median of 23 days (range: 11–24 days) after placement because of abundant granulation tissue formation. The Kaplan-Meier curves showed survival time for covered and uncovered stent implantations in patients with benign tracheobronchial stenosis (median 25 days vs 1339 days, *P* < 0.0001) (Fig. 1).

### Complications

No early complications were reported in 20/59 covered stents or in 32/72 uncovered stents. Covered stents were associated with more sore throats or chest pain (8.5% versus 28.81%, *P* = 0.036) when compared with the uncovered stents. There was no significantly different between the two groups in terms of severe cough or hemoptysis (25% versus 25.42%, *P* = 0.96; 16.67% versus 11.86%, *P* = 0.44, respectively).

**Fig. 2:**
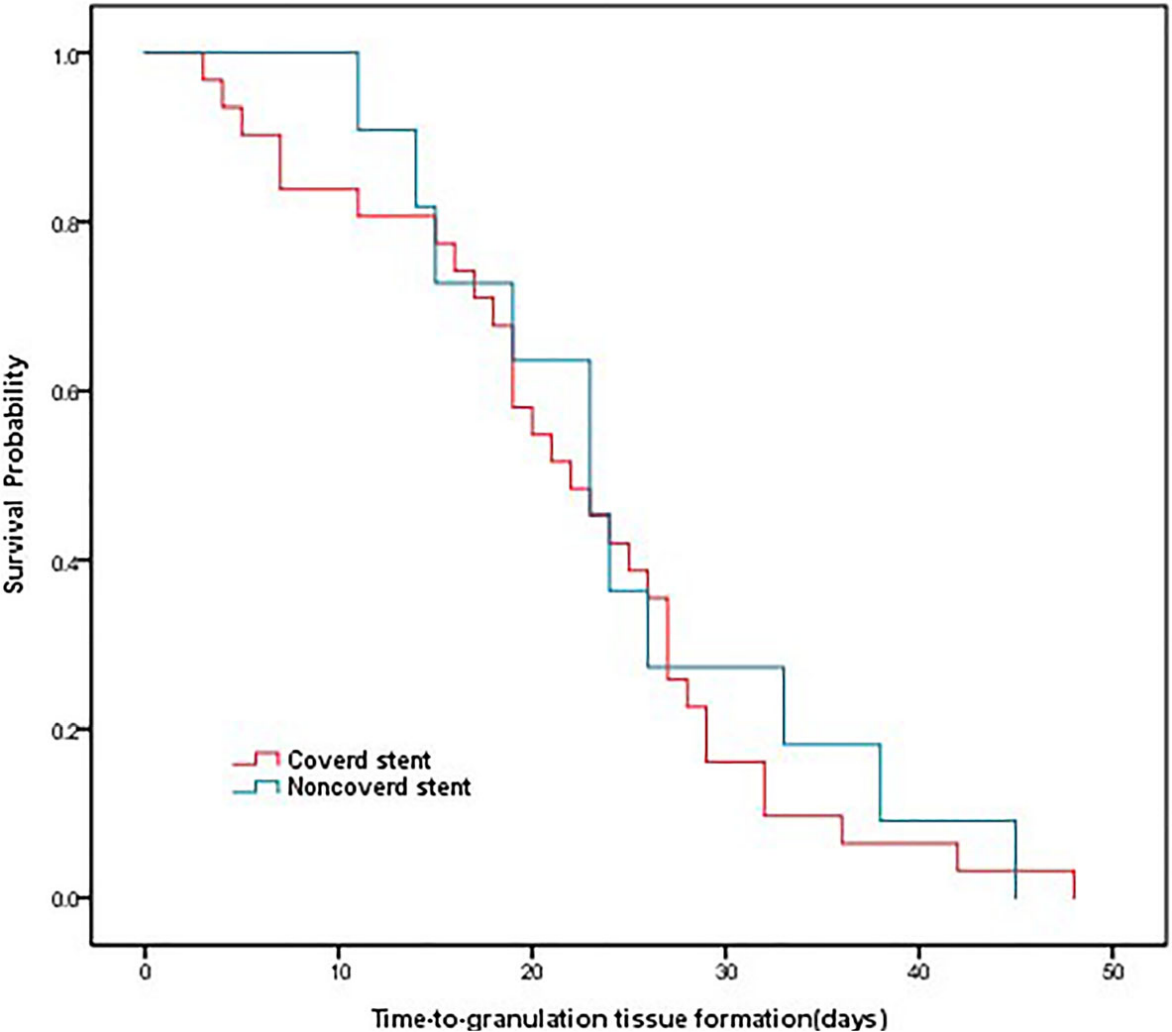
Time-to-granulation tissue formation curves Kaplan-Meier curves showing time-to-granulation tissue formation for covered and uncovered stents implantation in patients with benign tracheobronchial stenosis (median 22 days vs 23 days, *P* = 0.659)

Covered stents were associated with higher incidences of granulation tissue formation, sputum retention, and recurrent stenosis than were uncovered stents (major granulations: 15.25% versus 4.17%, *P* = 0.029; minor granulations: 37.29% versus 11.11%, *P* < 0.0001; sputum retention: 42.37% versus 18.06%, *P* = 0.02; and recurrent stenosis: 28.81% versus 9.72%, *P* = 0.005). The Kaplan-Meier curves showed time-to-granulation tissue formation for covered and uncovered stents implantation in patients with benign tracheobronchial stenosis (median 22 days vs 23 days, *P* = 0.659) (Fig. 2). There was no significantly different in stent migration or stent breakage between the two groups (11.86% versus 1.39%, *P* = 0.13; and 1.69% versus 0.00%, *P* = 0.27, respectively). No late complications were reported for 17/59 covered stents or 42/72 uncovered stents (28.81% versus 58.33%, *P* = 0.001). Stent complications are showed in Table 2.

**Table 2:** Stent (*n*) complications

Variable	Total	Covered stent	Uncovered stent	*P* value
Stents (*n*)	131	59	72	
Early complications				
Sore throat or chest pain	27 (20.61%)	17 (28.81%)	10 (13.89%)	*P* = 0.036
Severe cough	33 (25.19%)	15 (25.42%)	18 (25%)	*P* = 0.96
Hemoptysis	19 (14.50%)	7 (11.86%)	12 (16.67%)	*P* = 0.44
No early complications	52 (39.69%)	20 (33.9%)	32 (44.44%)	*P* = 0.22
Latter complications				
Major granulation (requiring stent replacement)	15 (11.45%)	9 (15.25%)	3 (4.17%)	*P* = 0.029
Minor granulation	30 (22.90%)	22 (37.29%)	8 (11.11%)	*P* < 0.0001
Sputum retention	38 (29.01%)	25 (42.37%)	13 (18.06%)	*P* = 0.02
Stent migration	8 (6.11%)	7 (11.86%)	1 (1.39%)	*P* = 0.13
Stent breakage	1 (0.76%)	1 (1.69%)	0 (0.00%)	*P* = 0.27
Recurrent stenosis	24 (18.32%)	17 (28.81%)	7 (9.72%)	*P* = 0.005
No latter complications	59 (45.04%)	17 (28.81%)	42 (58.33%)	*P* = 0.001

The values are expressed as number (percentage); Abbreviations: *p* value, covered vs uncovered stents

### Outcomes and follow-up

Technical success was accomplished in all but 5 patients in whom SEMS was deployed in an erroneous location requiring removal during the same bronchoscopy session. After SEMS placement (on average 2–3 days), 98 (84.48%; CI: 77.89–91.07) patients presented clinical improvement. Eighteen patients, 7 with severe pneumonia, 3 with chronic obstructive pulmonary disease, 3 with congestive heart failure, 3 with stent migration, 1 with lymphoma, and 1 with myasthenia gravis, showed no considerable clinical improvement (15.52%; 95% CI: 8.93–22.11) (Table 1).

**Fig. 3:**
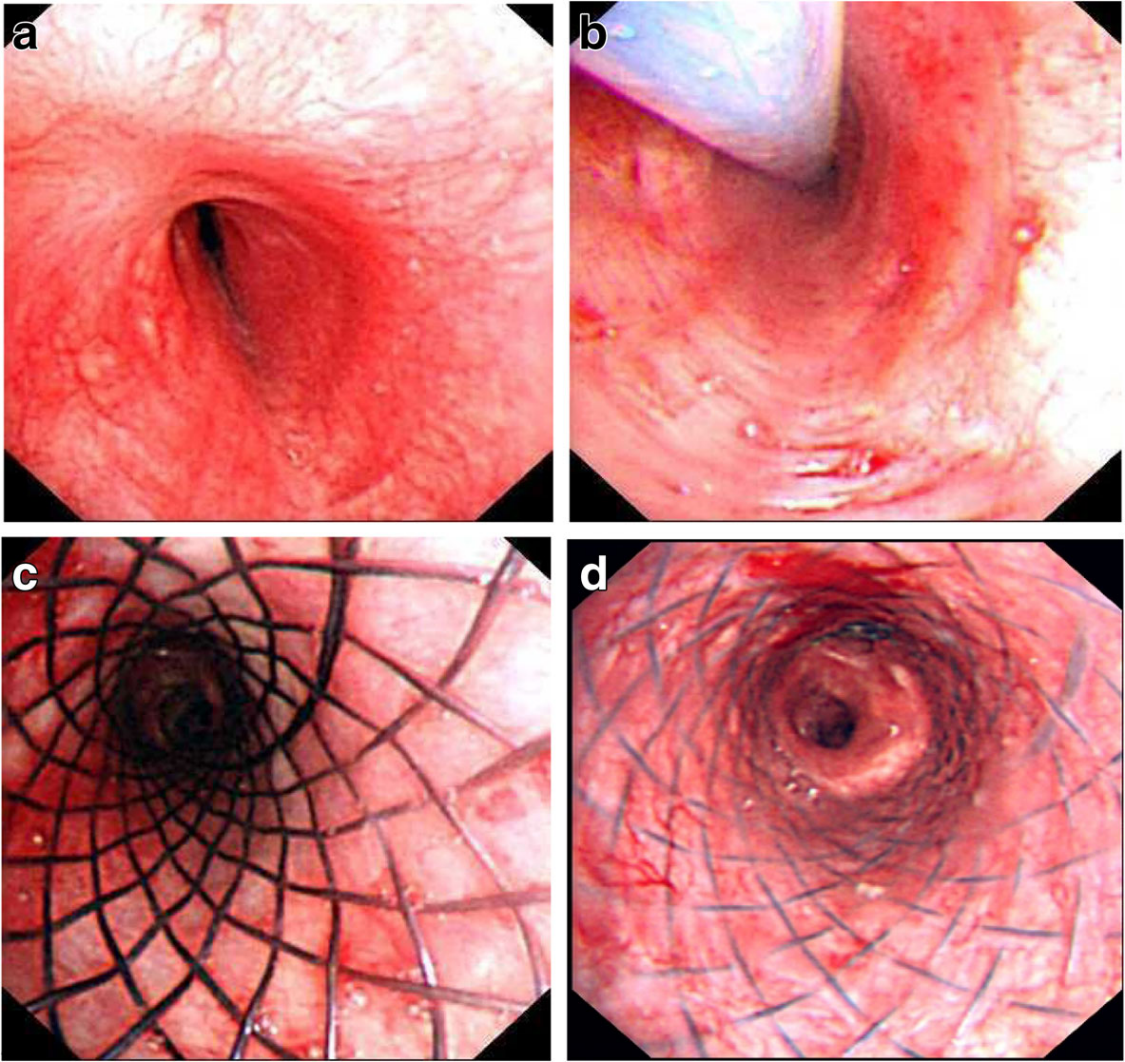
One successful patient report. A 33-year old woman with tuberculosis infection showed left main stem bronchial stenosis (**a**), and she underwent uncovered nitinol stent (14 × 30 mm) positioning in the narrow bronchus (**b** and **c**). Within the followed-up of seven years, the patient exhibited complete resolution. The last bronchoscope showed the stent was well attached with the bronchial wall and the lumen was clear (**d**)

Complete resolution was observed in 68 patients (66.67%; 95% CI: 57.52–75.8) at follow-up, 15 were still under interventional treatment, 8 had bronchial occlusions, 7 underwent surgery, 14 were lost to follow-up, and 4 died. Death was not associated to airway stenting in any of the 4 cases and was due to the primary disease: respiratory cycle failure secondary to chronic obstructive pulmonary disease (2), lymphoma (1), myasthenia gravis (1). For patients with both covered and uncovered metallic stents, the percentages of resolution were 53.34% (38.76–67.92%) for covered stents and 77.19% (66.3–88.08%) for uncovered stents. Figure 3 shows a 33-year-old woman with tuberculosis infection who underwent uncovered nitinol stent positioning in the narrow bronchus. This patient achieved the resolution within the 7-year follow-up. Outcomes and stent types are reported in Table 3.

**Table 3:** Relationship between stent type and definitive outcomes in all patients (*n*)

	Total		Covered stent		Uncovered stent	
	*n*	%	*n*	%	*n*	%
Resolution	68	66.67% (57.52–75.82%)	24	53.34% (38.76–67.92%)	44	77.19% (66.3–88.08%)
Still under treatment	15	14.71% (7.84–21.58%)	6	13.33% (3.4–23.26%)	9	15.79% (6.32–25.26%)
Bronchial occlusion	8	7.84% (2.62–13.06%)	6	13.33% (3.4–23.26%)	2	3.51% (−1.27–8.29%)
Surgery^a^	7	6.86% (1.95–11.77%)	7	15.56% (4.97–26.15%)	0	0%
Death^b^	4	3.92% (0.15–7.69%)	2	4.44% (− 1.58–10.46%)	2	3.51% (−1.27–8.29%)
Total	102	100%	45	100%	57	100%
Lost to follow-up	14	–	5	–	9	–
Total	116	–	50	–	66	–

The values are expressed as percentage (CI)

^a^Tracheoplasty 1, Lobectomy 3, Resection and anastomosis 3

^b^All cases death was not associated to airway stenting but to the primary disease: respiratory cycle failure secondary to chronic obstructive pulmonary disease (2), lymphoma (1), myasthenia gravis (1)

### Subgroup analysis

Based on the common causes of benign airway stenosis, we divided patients into two groups: 1) post-inflammatory stenosis (*n* = 56), including patients with tuberculosis and fungal infection; and 2) post-traumatic stenosis (*n* = 57), which includes patients with tracheotomy, endotracheal tube, bronchial repair and tracheoesophageal fistula repair. The basic conditions of patients, rates of clinical improvement, common complications of stenting, and outcomes were compared. Patients with post-inflammatory stenosis had high rates of minor granulation formation and were more prone to developing bronchial occlusions than patients with post-traumatic stenosis (25% versus 15.79%, *P* = 0.024; 15.69% versus 0.00%, *P* = 0.004, respectively). Patients with post-traumatic stenosis required significantly more reinterventions than did patients with post-inflammatory stenosis (57.89% versus 32.14%, *P* = 0.008). No substantial difference in clinical improvement and resolution was observed between the two groups. The results of subgroup analysis are presented in Table 4.

**Table 4:** Analysis of stricture causes

Values	Post-inflammatory stenosis (*n* = 56)	Post-traumatic stenosis (*n* = 57)	*P* value
Male/female	16/40	38/19	
Age (mean ± SD)	32.98 ± 9.64	43.77 ± 14.78	*P* = 0.134
Coverd/uncovered stent (*n*)	21/35	28/29	
Clinical improvement	45 (80.36%)	49 (85.96%)	*P* = 0.21
Major granulation (requiring stent replacement)	3 (5.36%)	9 (15.79%)	*P* = 0.072
Minor granulation	14 (25%)	9 (15.79%)	*P* = 0.024
Recurrent stenosis	6 (10.71%)	13 (22.81%)	*P* = 0.086
Further interventions			
Balloon dilatation	13 (23.21%)	15 (26.32%)	*P* = 0.73
Electrocautery	9 (16.07%)	20 (35.09%)	*P* = 0.21
Cryotherapy	2 (3.57%)	8 (14.04%)	*P* = 0.05
APC	1 (1.79%)	4 (7.02%)	*P* = 0.157
Total	18 (32.14%)	33 (57.89%)	*P* = 0.008
Outcomes			
Resolution	36 (70.59%)	30 (61.22%)	*P* = 0.323
Still under treatment	3 (5.88%)	11 (22.45%)	*P* = 0.017
Bronchial occlusion	8 (15.69%)	0 (0.00%)	*P* = 0.004
Surgery	3 (5.88%)	5 (10.2%)	*P* = 0.426
Death	1 (1.96%)	3 (6.12%)	*P* = 0.288

The values are expressed as mean ± SD or number (percentage); Abbreviations: *p* value, Post-inflammatory stenosis vs Post-traumatic stenosis, *APC* argon plasma coagulation

## Discussion

The present study shows a single institution’s experience in the management of tracheobronchial obstruction with SEMSs in benign diseases. Several reports dedicated to SEMSs have been published, but none included such a large quantity of patients or long-term follow-up [8–20].

Post-tuberculosis tracheobronchial stenosis (PTTS) and post-traumatic stenosis (after intubation or tracheostomy) are considered to be the most common two causes of benign airway stenosis [21, 22]. Our study showed similar results in that the main causes of stricture were PTTS (47.41%) and post-traumatic stenosis (45.69%). In 55 patients with PTTS, 36 SEMSs were implanted in the left main stem bronchus, which indicated bronchial tuberculosis amenable to stenting and frequently occurred on the left side. Compared with the previous largest series [4, 9], our study included a greater quantity of patients and showed a higher rate of clinical improvement (84.48%, *n* = 116 versus 62.9%, *n* = 69, and 76.7%, *n* = 72 respectively). Technical success was implemented in 100% of individuals, and clinical success was achieved in 66.67% (95% CI: 57.52–75.82%). None of the deaths stated in our study were associated to stent implantation or its longevity in the airway. Madan et al. [23] reported a multicenter experience with the placement of metallic Y stents, but all patients in that study had malignant airway obstruction or airway fistulization near the tracheal carina. In our study, Y stents were inserted in one patient with relapsing polychondritis.

The Food and Drug Administration recommendation in 2005 stated that the use of metallic stents to treat benign tracheal stenosis should be considered only after thorough exploration of all other treatment options, like surgical treatment or insertion of silicone stents [24]. However, silicone stents were not available in some developing countries in previous years. Compared to silicone stents, SEMSs have their advantages, such as lower migration rate, better conformation to irregular airways, greater cross-sectional airway diameter because of thinner wall construction, in favor of mucociliary clearance due to epithelialization within the stent, and a greater ease of placement [4]. Moreover, SEMSs can be successfully placed with a flexible bronchoscope under conscious sedation and local anesthesia [18, 25].

Given the specific of SEMSs, early complications after SEMS implantation include sore throat, chest pain, death, severe cough, hemorrhage, and airway perforation [10, 11, 26–28]. In this series, 19 patients had minor bleeding during stent placement that was successfully controlled with bronchoscopy compression. Compared to the uncovered stents, covered stents showed a higher rate of sore throat or chest pain, which probably resulted from the self-expanding force that facilitates the stent to attach directly to the airway wall. With respect to late complications, granulation tissue formation had been reported around stents with a frequency of 12–46% [4, 5, 18]. In our study, there was a relatively high incidence of granulation tissue formation (34.35%) during the median period of 1276 days, which may be attributed to larger quantity of patients and longer follow-up time. Previous studies have shown that the incidence and degree of granulation tissue hyperplasia may be related to a multitude of factors, such as the pressure of the stent on the wall, the friction of the airway wall, and local airway infection [29]. However, the etiopathogenesis of granulation in SEMSs is still unclear, and more data and prospective studies are needed.

The total complication frequencies in our research are analogous to those reported in previous studies [5, 7, 9, 21]. SEMS-related hyperplastic tissue formation and restenosis caused by hypertrophic scar tissue can be managed with various flexible bronchoscopic interventions when necessary [9]. In our study, time-to-granulation tissue formation of covered and uncovered stents was about twenty days. SEMS-related complications should be managed promptly after stents insertion. These complications require interventions by experienced bronchoscopists who have mastered interventional techniques such as balloon dilation, electrocautery, and other therapies.

The optimal duration for placement of the temporary metallic stent still remains controversial. Chen et al. suggested that the optimal placement time for a covered stent is between 2 and 6 months [30]. In our study, the survival time of covered and uncovered stents was a median of 25 and 1339 days, respectively. Rodriguez et al. indicated uncovered metal stents have a theoretic advantage of neo-epithelialization that stent could incorporate into the airway wall [31]. However, the neo-epithelialization could also be a cause for concern if granulation tissue produces a recurrent obstruction inside the stent [26]. Our records did not demonstrate this problem because the stent had been selected based on the type of disease before being implanted. Most patients in our study had airway malacia or cicatricial stenosis; thus, uncovered stents were selected. There were 44 (78%) patients who maintained patency with neo-epithelialized stents out of the 66 total patients who had uncovered stents inserted during a median follow-up period of 1276 days (range: 2–4263 days).

According to our experience, sedation and local anesthesia are adequately prepared before bronchoscopy. Sedation with intravenous midazolam (5 mg) and a local anaesthetic with 2% xylocaine solution were administrated prior to bronchoscopy [6]. During the operation, if the patient has a cough or movement, the 2% xylocaine was sprayed in the trachea. Because non-availability of fluoroscopic guidance, we used a guidewire to provided direct visualization of stent placement, which ensures the accuracy of stent position. While, the technique required an experienced bronchoscopist to ensure safety of patient.

There were several limitations to our study. First, it was a retrospective study of a single center, the outcome measures were not well-defined and there was the possibility for missing data from the retrospective review. Therefore, future prospective controlled studies are needed. Second, several of our patients were lost to follow-up and could not be evaluated for long-term success. Third, it may be difficult to clarify the complex factors contributed to complications, even though our study reported a higher frequency of granulation formation after SEMS deployment in patients with benign tracheobronchial stenosis. Consequently, long-term prospective multicenter studies are needed to clearly define clinical indications for airway stenting and its therapeutic value in selected patients.

## Conclusion

The placement of a self-expanding nitinol stent achieved most clinical improvement among patients in our study. Implantation of SEMSs for treatment of benign airway stenosis was reliable. The high rate of resolution can be achieved with further intervention. Although SEMS-related complications are inevitable, adequate endotracheal measures can be used to address them. Our experience verifies that using permanent SEMSs for benign tracheobronchial stenosis is effective and safe for the majority of patients in a long-term follow-up.

## Abbreviations

APC: Argon plasma coagulation
CI: Confidence interval
CT: Computed tomography
LMSB: Left main stem bronchus
LT: Lower trachea
MT: Middle trachea
PTTS: Post-tuberculosis tracheobronchial stenosis
RMLB: Right middle lobar bronchus
RMSB: Right main stem bronchus
SEMSs: Self-expandable metallic stents
UT: Upper trachea
